# Design and Performance Study of an Ultrasonic Synthetic Jet Piezoelectric Pump Based on Multi-Level Structural Optimization

**DOI:** 10.3390/mi17090994

**Published:** 2026-08-23

**Authors:** Zixin Chen, Yilin Li, Wenjun Li, Keqiang Yue, Ruixue Li

**Affiliations:** School of Electronic Information, Hangzhou Dianzi University, No. 1158, Avenue 2, Qiantang District, Hangzhou 310018, China; 242040132@hdu.edu.cn (Z.C.); liwenjun@hdu.edu.cn (W.L.); kqyue@hdu.edu.cn (K.Y.); 42949@hdu.edu.cn (R.L.)

**Keywords:** synthetic jet, piezoelectric pump, micropump, micro-electro-mechanical system (MEMS) device, fluid–structure interaction simulation, optimization of resonant structures

## Abstract

The present work presents a new synthetic jet piezoelectric pump designed to address the airflow delivery needs arising from the increasing power density of high-performance microelectronics. Traditional miniaturized cooling techniques suffer from low efficiency, bulky size, and high cost, while microfluidic cooling has emerged as a vital chip thermal management method with outstanding miniature heat removal capacity. We systematically designed the vibration mode and pump structure, adopting the sixth-order resonant frequency as the operating frequency. A dual resonant layer with stiffness-guided fixed boundaries was employed to enhance vibration efficiency and energy conversion, together with an optimized flow channel layout and parametric design. Experiments conducted under 35 V square-wave excitation demonstrate that the 20 mm × 20 mm × 2.5 mm pump delivers a flow rate of 1.6 L/min and a back pressure of 2.7 kPa. This work provides a feasible technical route for large-scale airflow delivery applications of synthetic jet piezoelectric pumps, with potential for thermal management in microelectronic devices, while balancing excellent performance and low manufacturing cost.

## 1. Introduction

As integrated circuit technology continues to advance, chip computing power has increased significantly while chip dimensions continue to shrink [1]. The resulting sharp increase in power density has led to a substantial rise in heat generation, severely limiting device efficiency and reliability [2]. This issue is particularly acute in high-performance microelectronic devices [3]. Traditional air and liquid cooling technologies suffer from low efficiency, large size, and high cost in miniaturized applications, making them inadequate for meeting current heat dissipation demands [4]. Their adaptability is further restricted in space-constrained systems [5]. More efficient heat dissipation solutions are therefore urgently needed [6]. Microfluidic heat dissipation systems, owing to their ability to remove heat efficiently at small scales, have emerged as a key approach in chip thermal management [7], and their excellent performance under local high-heat-flux conditions has been widely validated [8]. In such systems, the micropump serves as the core driving component; its performance therefore directly determines the overall heat dissipation efficiency [9]. Previous studies have shown that the flow rate and pressure differential of micropumps play a decisive role in heat dissipation performance. However, existing micropumps generally suffer from low flow rates, high power consumption, and complex fabrication processes, which limit their widespread adoption [10]. High power consumption is particularly problematic in battery-powered devices, and complex manufacturing processes markedly increase production costs [11]. Developing micropumps that combine low power consumption, high flow rate, and low manufacturing cost therefore represents a critical technical challenge [12]. Overcoming this bottleneck will significantly advance the development of miniaturized heat dissipation systems [13] and is expected to play a key role in fields such as mobile devices and wearable electronics [14].

Recent advances in micro-electro-mechanical system (MEMS) technology have significantly accelerated the development of miniaturized heat sinks [15]. The increasing maturity of MEMS fabrication has enabled the production of complex microchannel structures [16], thereby broadening the application scope of compact heat sinks [17]. The inverse piezoelectric effect of piezoelectric materials has been harnessed in synthetic jet piezoelectric pumps to drive airflow, generating high-flow-rate, high-back-pressure synthetic jets with potential for efficient thermal management [18,19]. This technology is particularly well suited to applications with limited installation space [20], and its distinctive flow characteristics contribute to enhanced heat transfer performance [21]. By combining the superior electromechanical conversion capability of piezoelectric ceramics with the flow control advantages of synthetic jets, high-performance airflow delivery can be achieved within compact dimensions. Moreover, the rapid response of piezoelectric materials further enhances the dynamic performance of the system [22], offering a promising airflow delivery solution with potential thermal management applications in next-generation high-power-density electronic devices [23,24].

Significant progress has been reported in the study of synthetic jet piezoelectric pumps. Jalilvand et al. developed a dual-cooling-jet synthetic jet actuator capable of generating airflow velocities exceeding 7 m/s under specific conditions, representing a 3.5-fold improvement over the single-jet actuator [25]. Wang et al. designed an ultrasonic injection piezoelectric pump to enhance gas delivery using a piezoelectric actuator, achieving a flow rate of up to 390 mL/min at 80 Vpp and 20.4 kHz [26]. Liu et al. reported a maximum output flow rate of 2.79 L/min at 150 Vpp and 3.15 kHz sinusoidal excitation using an optimized pump structure, and subsequently developed a control system for electronic cooling [27]. Liu and Zhu further developed two synthetic jet direct-injection piezoelectric pump configurations, namely single- and double-chamber versions [28]; under 150 Vpp, 2.38 kHz sinusoidal excitation, their output flow rates reached 2.1 and 2.43 L/min, respectively, providing a basis for further development in electronic cooling. Li et al. developed a piezoelectric pump with a petal-shaped inlet channel, achieving a maximum flow rate of 1.8929 L/min at 100 V and 3.9 kHz for the unoptimized design, which increased to 3.0088 L/min at 100 V and 3.7 kHz after optimization, corresponding to a 58.95% performance improvement [17]. Wang et al. conducted structural optimization that yielded an 86.8% increase in the output flow rate of a piezoelectric pump, reaching 1037 mL/min [29]; after further optimization of the driving waveform, the flow rate increased to 1343.9 mL/min. When two chambers of a dual-chamber ultrasonic synthetic jet piezoelectric pump operated in parallel, the flow rate reached 1912.68 mL/min, although the overall thickness was 6.5 mm.

In summary, although significant progress has been made in the performance optimization of synthetic jet piezoelectric pumps, they still face the following common challenges: (1) most studies require a high driving voltage (≥80 Vpp) to achieve large output flow rates, which is unfavorable for low-power, portable applications; and (2) device thickness generally exceeds 5 mm, which cannot satisfy the requirements of increasingly compact integration spaces. To address these issues, this paper proposes a new piezoelectric pump structure with an emphasis on the systematic design of the vibration mode and pump body configuration. The proposed structure operates at the sixth-order resonant frequency and employs a piezoelectric ceramic driving disk with a radius of 5.4 mm, which significantly increases the vibration amplitude while maintaining a high operating frequency. A configuration combining a dual resonant layer and a stiffness-guided fixed boundary is proposed to effectively improve the vibration efficiency and energy conversion performance of the system. The flow channel layout design and parametric optimization are also carried out. Experimental results demonstrate that under 35 V square-wave excitation, the proposed piezoelectric pump delivers an output flow rate of 1.6 L/min and a back pressure of 2.7 kPa within a compact size of 20 mm × 20 mm × 2.5 mm. The present work offers a novel technological pathway for the large-scale application of synthetic jet piezoelectric pumps in airflow delivery for microelectronic devices, with potential extension to thermal management, while achieving a favorable balance between performance and cost.

## 2. Device Structure and Operating Mechanism

### 2.1. Overall Structural Design of Pump Body

**Figure 1:**
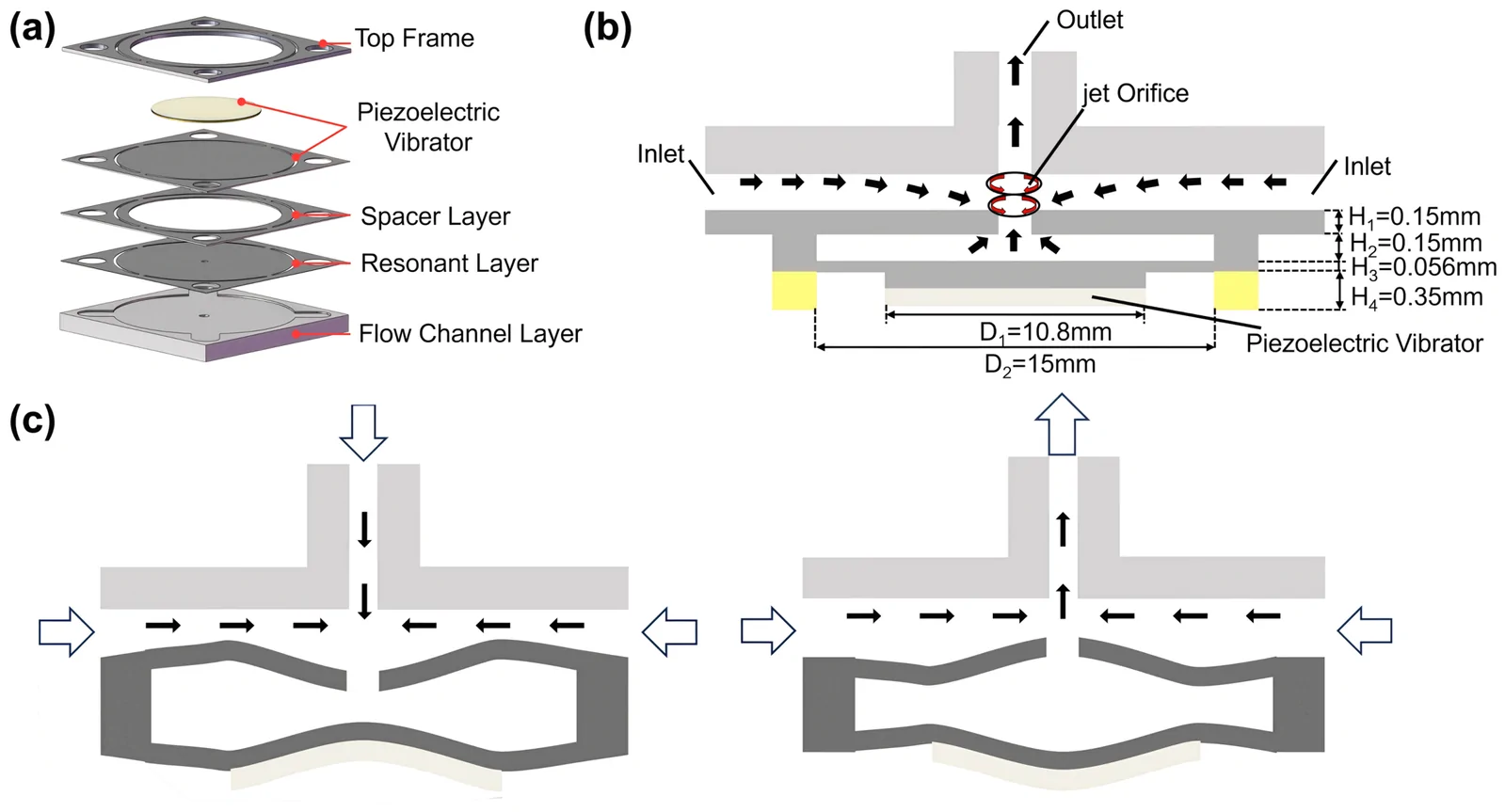
The piezoelectric pump structure: (**a**) Three-dimensional structure; (**b**) Two-dimensional structure; (**c**) Working process.

The synthetic jet piezoelectric pump developed in this work is a gas-driven device based on the piezoelectric vibration effect. It converts input electrical signals into mechanical vibrations and further transforms vibration energy into gas kinetic energy, thereby achieving directional gas transport. As shown in Figure 1a, the pump is precisely assembled from core components including the top frame, piezoelectric vibrator, diaphragm, resonant layer, and flow channel layer. The overall design balances high output flow rate with low energy loss to improve the efficiency and practical applicability of the device.

The piezoelectric vibrator is the core driving unit of the system. It operates in the ultrasonic frequency range (≥20 kHz) and generates periodic mechanical vibrations under alternating voltage excitation. The flow channel layer performs a flow-rectifying function, and its structural parameters exert a significant influence on system performance. As shown in Figure 1b, the jet orifice is located at the center of the jet plate and is connected to the pump chamber. It guides the gas to be efficiently ejected from the pump chamber through the jet orifice, thereby forming a directional flow and providing a reliable structural basis for gas-driven applications.

### 2.2. Working Principle of Piezoelectric Actuation and Synthetic Jet Formation

The working principle of the pump is illustrated in Figure 1c. When excited by a square-wave signal matching its resonant frequency, the piezoelectric vibrator undergoes periodic deformation via the inverse piezoelectric effect, driving the pump chamber through alternating suction and discharge cycles. In the negative half-cycle, the piezoelectric ceramic contracts, causing the center of the vibrator to deflect toward the chamber. As a result, the pump chamber volume increases, generating negative pressure that draws external gas into the chamber. During the positive half-cycle, the piezoelectric ceramic expands, and the vibrator center deflects away from the chamber, reducing the chamber volume and increasing the internal pressure. Consequently, gas is ejected at high velocity through the jet orifice. The jet outlet velocity reaches its maximum when the vibrator is at the position farthest from the jet orifice.

In accordance with the synthetic jet formation principle, the ejected airflow possesses sufficient directional momentum to propagate forward and is not re-entrained into the chamber during the suction phase. Through the continuous periodic vibration of the piezoelectric vibrator, stable unidirectional gas transport is ultimately achieved.

### 2.3. Criterion for Stable Synthetic Jet Formation

To ensure stable synthetic jet formation, the gas exit velocity near the jet orifice during the suction process must exceed the velocity at which fluid would be re-entrained into the chamber. This condition prevents backflow and ensures effective generation of synthetic jets. Whether the system can produce stable synthetic jets can be predicted using the fluid Reynolds number (Re) and Stokes number (St). The criterion is expressed as follows:

Re/St^2^ = U_0_/(ω · d) > C (1)

where St represents the ratio of unsteady inertial to viscous effects, and Re reflects the ratio of inertial to viscous forces. U_0_ is the average jet velocity, ω is the angular vibration frequency, ν is the fluid kinematic viscosity, and d is the jet orifice diameter. The constant C depends on the flow dimensionality: C = 1 for two-dimensional conditions and C = 0.16 for three-dimensional conditions. When this inequality is satisfied, the system is capable of generating stable synthetic jets. This criterion comprehensively captures the coupled effects of driving voltage (through its influence on flow amplitude and frequency), material elastic modulus (which determines structural stiffness and natural frequency), and chamber dimensions (which affect fluid impedance and structural dynamics) on jet formation. In this study, the system operates under three-dimensional conditions, and the experimental calculation yields Re/St^2^ = 1.31 > 0.16, confirming that the design parameters satisfy the criterion for stable synthetic jet formation.

## 3. Modal Analysis and Fluid–Structure Interaction Simulation of the Piezoelectric Vibrator

### 3.1. Sixth-Order Modal Simulation Analysis of Piezoelectric Vibrator

The piezoelectric vibrator is the core driving unit of the synthetic jet piezoelectric pump. Its vibration mode characteristics determine the rate of change in pump chamber volume and gas transport capability, thereby directly governing the output flow rate and back pressure of the system. To achieve optimal output performance under a constant driving voltage, a modal simulation analysis of the piezoelectric vibrator was conducted in this study.

**Figure 2:**
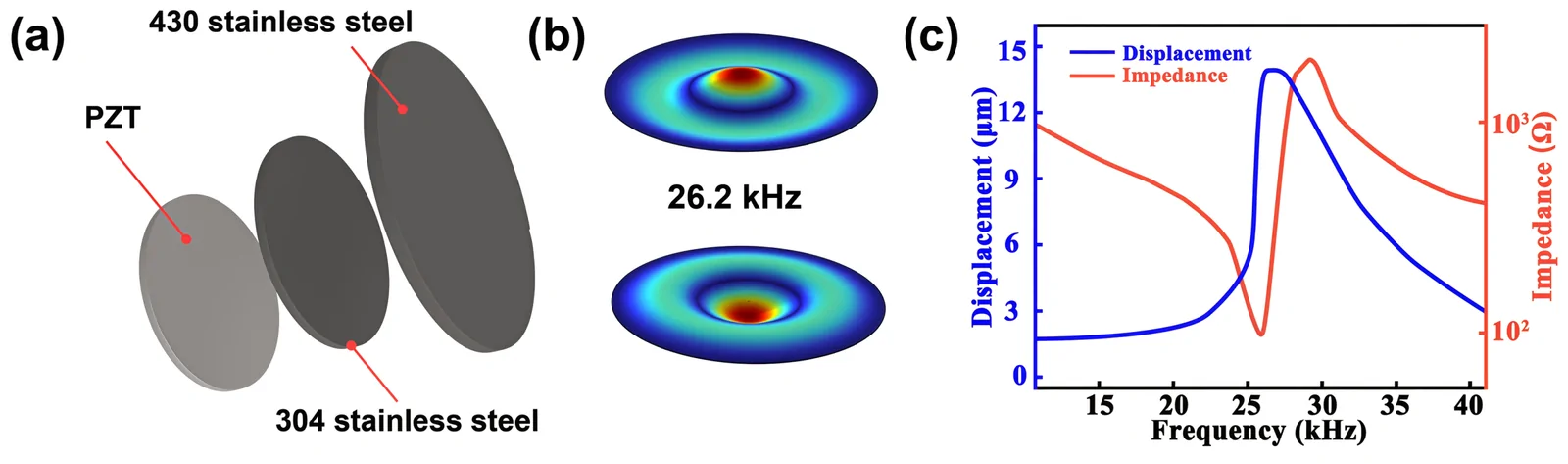
Structural and modal characteristics of piezoelectric vibrator: (**a**) Schematic diagram showing the composite structure of piezoelectric vibrator; (**b**) Simulation of the sixth-order mode shape; (**c**) Impedance/amplitude–frequency characteristic curves of the piezoelectric vibrator.

The piezoelectric vibrator adopts a three-layer composite structure formed by bonding a PZT-4H piezoelectric ceramic layer, a stainless steel 304 reinforcement layer, and a stainless steel 430 diaphragm layer, as shown in Figure 2a. The dimensions of each layer are as follows: PZT-4H piezoelectric ceramic (Ø10.4 mm × 0.2 mm), stainless steel 304 reinforcement layer (Ø10.8 mm × 0.15 mm), and stainless steel 430 diaphragm (Ø15 mm × 0.45 mm).

PZT was selected as the piezoelectric material due to its high piezoelectric strain coefficient (d_33_ ≈ 289 pC/N), high electromechanical coupling factor, well-established manufacturing process, and low cost. These properties make PZT the most widely used piezoelectric ceramic for actuator applications requiring large displacement at ultrasonic frequencies.

Modal simulations over the range of 10–40 kHz revealed that the sixth-order mode (26.2 kHz) exhibits an axisymmetric vibration pattern characterized by localized vibration in the form of a nodal ring. As shown in Figure 2b, the vibration energy is concentrated at the vibrator center, which reduces energy dissipation at the boundaries and improves the electromechanical conversion efficiency.

An impedance analyzer was used to characterize the piezoelectric vibrator, and the impedance and central vibration displacement curves were obtained (Figure 2c). The piezoelectric vibrator exhibited its lowest impedance at the resonant frequency of 26.2 kHz, where the peak central vibration displacement reached 14.1 μm. At this frequency, the electromechanical energy conversion efficiency was maximized, in agreement with the simulation results. The quality factor of the piezoelectric vibrator, determined from the impedance curve (Figure 2c) as Q = f_0_/Δf_−3_ᵈᴱ, is approximately 103, indicating a moderately high resonance sharpness suitable for efficient narrowband operation. Therefore, the sixth-order resonant mode was selected as the operating mode, enabling large vibration amplitudes at high frequencies and satisfying the output requirements of high flow rate and high back pressure.

### 3.2. Transient Fluid–Structure Interaction Simulation of Flow Field Inside Pump Chamber

#### 3.2.1. Numerical Model Setup

The numerical simulations were performed using COMSOL Multiphysics 6.3 (COMSOL Inc., Stockholm, Sweden). The modal analysis of the piezoelectric vibrator employed the built-in Piezoelectric Effect multiphysics coupling interface, which automatically couples the Solid Mechanics and Electrostatics modules. The transient flow field simulations utilized the Laminar Flow module with a moving mesh (ALE) formulation for fluid–structure interaction. The piezoelectric vibrator was analyzed in the frequency domain using the eigenfrequency solver to determine the resonant modes, as described in Section 3.1. The fluid flow within the pump chamber was modeled using the transient incompressible Navier–Stokes equations with the laminar flow assumption (ρ = 1.205 kg/m^3^, μ = 1.81 × 10^−5^ Pa·s).

The fluid–structure interaction was implemented using a two-way coupling scheme with an arbitrary Lagrangian–Eulerian (ALE) moving mesh formulation. A two-dimensional axisymmetric computational model was constructed with approximately 40,653 triangular and quadrilateral elements, with local mesh refinement applied near the jet orifice and along the wall boundaries to adequately resolve the high-velocity-gradient regions. The boundary conditions were as follows: (1) zero-gauge pressure at the inlet opening and jet orifice outlet, (2) no-slip condition on all solid walls of the pump chamber, (3) prescribed displacement on the piezoelectric vibrator surface obtained from the sixth-order modal analysis (Section 3.1), and (4) axisymmetric condition along the central axis. The segregated solver with a relative tolerance of 10^−3^ was employed, and the discretized linear systems were solved using the PARDISO direct solver.

A uniform time step of T/25 (≈1.53 μs, where T = 1/26.2 kHz ≈ 38.2 μs) was adopted for the transient simulations. Each simulation was run for 100 cycles to allow the flow field to reach a periodic steady state, at which point the displacement, chamber pressure, and inlet/outlet flow rate waveforms exhibited negligible cycle-to-cycle variation. Data from the subsequent 10 stable cycles were used to compute the cycle-averaged flow rate. The cycle-averaged flow rate was determined from the cumulative inlet and outlet flow curves by taking the net volume increment over the 10 stable cycles and dividing by the total time duration.

The material properties used in the numerical model are summarized in Table 1.

#### 3.2.2. Flow Field Evolution

**Figure 3:**
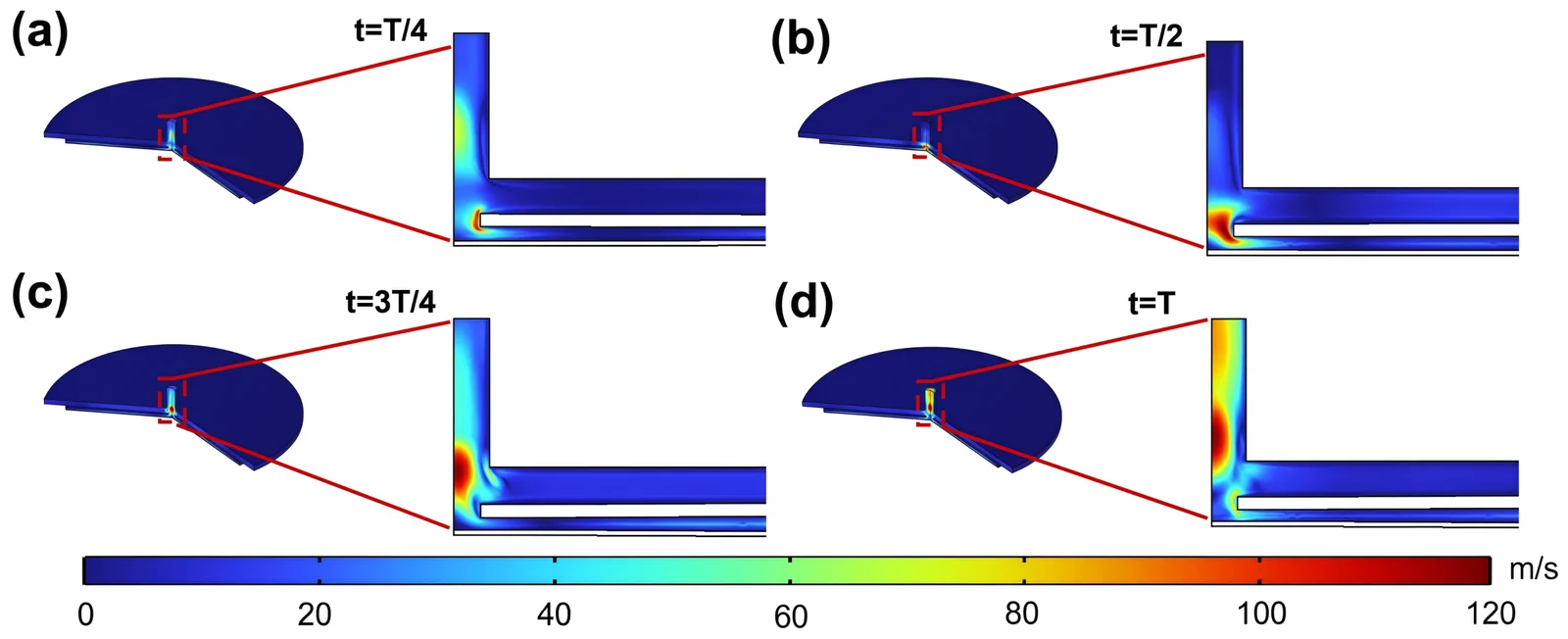
Flow field analysis of the synthetic jet over one cycle (**a**) Simulated flow field at 0–0.25 T (**b**) Simulated flow field at 0.25 T–0.5 T (**c**) Simulated flow field at 0.5 T–0.75 T (**d**) Simulated flow field at 0.75 T–1 T.

To reveal the internal flow features of the ultrasonic synthetic jet piezoelectric pump, a two-dimensional axisymmetric model was constructed and transient numerical simulations were performed. Figure 3 illustrates the evolution of the flow field throughout a full cycle, clearly demonstrating the generation and development mechanisms of the vortex structure. The process can be divided into four typical stages throughout a full cycle, clearly demonstrating the generation and development mechanisms of the vortex structure. The process can be divided into four typical stages:

As shown in Figure 3a, during the suction stage (0–0.25 T), the piezoelectric vibrator center deflects away from the jet orifice, increasing the pump chamber volume and generating negative pressure. External gas is drawn into the chamber through both the inlet channel and the jet orifice, while a high-velocity shear flow develops near the orifice due to the throttling effect. As shown in Figure 3b, during the first half of the discharge stage (0.25 T–0.5 T), the piezoelectric vibrator reverses its motion, expelling gas from the chamber. The shear layer rolls up at the orifice and begins to form a vortex ring structure. In the second half of the discharge stage (0.5 T–0.75 T), the jet velocity reaches its peak, and the vortex ring becomes fully developed and detaches from the orifice, as shown in Figure 3c. Finally, during the subsequent suction stage (0.75 T–1 T), as shown in Figure 3d, the detached vortex ring propagates away from the jet orifice under self-induction, enabling directional transport of the synthetic jet without interference from the following suction process.

## 4. Multi-Level Innovative Design of a High-Performance Pump Structure

### 4.1. Design and Frequency Mismatch Optimization of the Dual-Resonant-Layer Coupled Pump Chamber

**Figure 4:**
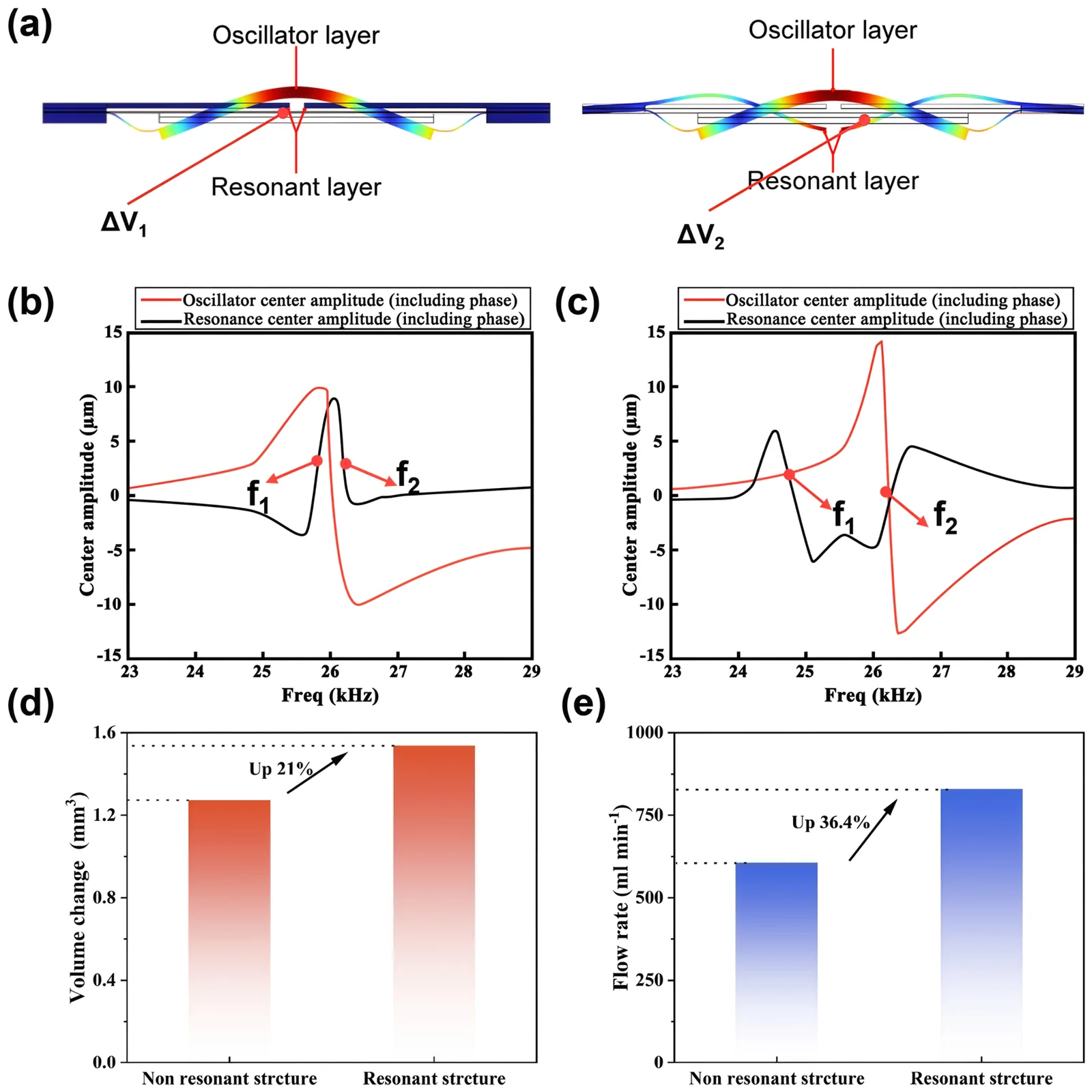
Working mode and vibration characteristics of the coupled pump chamber: (**a**) Comparison between the single-vibrator vibration mode and double-wall out-of-phase vibration mode; Frequency response curves of coupled system before (**b**) and after (**c**) optimization; (**d**) Comparison of chamber volume change before and after optimization; (**e**) Comparison of flow rate before and after optimization.

To enhance the output performance of the single-jet-orifice piezoelectric pump, a resonant structure was introduced into the bottom frame, building upon the conventional piezoelectric vibrator–middle-frame configuration to construct a coupled pump chamber capable of out-of-phase vibration. The synthetic jet performance is directly determined by the efficiency of pump chamber volume variation (ΔV). Theoretical analysis indicates that the rate of chamber volume change is maximized when the upper and lower walls of the pump chamber (the piezoelectric vibrator and the resonant layer) vibrate in opposite phases. Figure 4a clearly illustrates the fundamental difference in operating modes between non-resonant single-wall vibration and resonant out-of-phase dual-wall vibration.

However, the introduction of a resonant structure also gives rise to modal interference. When the natural frequency of the resonant layer coincides with the operating frequency of the piezoelectric vibrator, the system excites two adjacent resonance peaks (f_1_ and f_2_), corresponding to the in-phase and out-of-phase modes, respectively. This frequency overlap can lead to reduced vibration amplitude and unstable responses. Moreover, during practical operation, thermally induced frequency drift may trigger modal transitions, seriously compromising output reliability. To address this issue, a frequency mismatch optimization strategy was proposed. By appropriately reducing the thickness of the resonant layer, its natural frequency was adjusted to be slightly lower than the operating resonant frequency of the piezoelectric vibrator. Figure 4b and Figure 4c present the frequency responses before and after optimization, respectively. These results demonstrate that the optimization effectively increased the frequency separation between f_1_ and f_2_, thereby broadening the stable operating bandwidth around the out-of-phase mode (f_2_).

The optimized system exhibits substantial performance improvement. A comparison of Figure 4d,e shows that the resonant structure achieves a higher volume change rate and a better frequency response in the out-of-phase mode. The simulation results confirm that, after optimization of the resonant structure, the chamber volume change rate increased from approximately 1.26 mm^3^ to 1.53 mm^3^, representing a 21% increase. Furthermore, the peak flow rate increased from 607.61 mL/min to 828.78 mL/min, representing a 36.4% improvement.

### 4.2. Design of the Stiffness-Guided Fixed Boundary Structure

**Figure 5:**
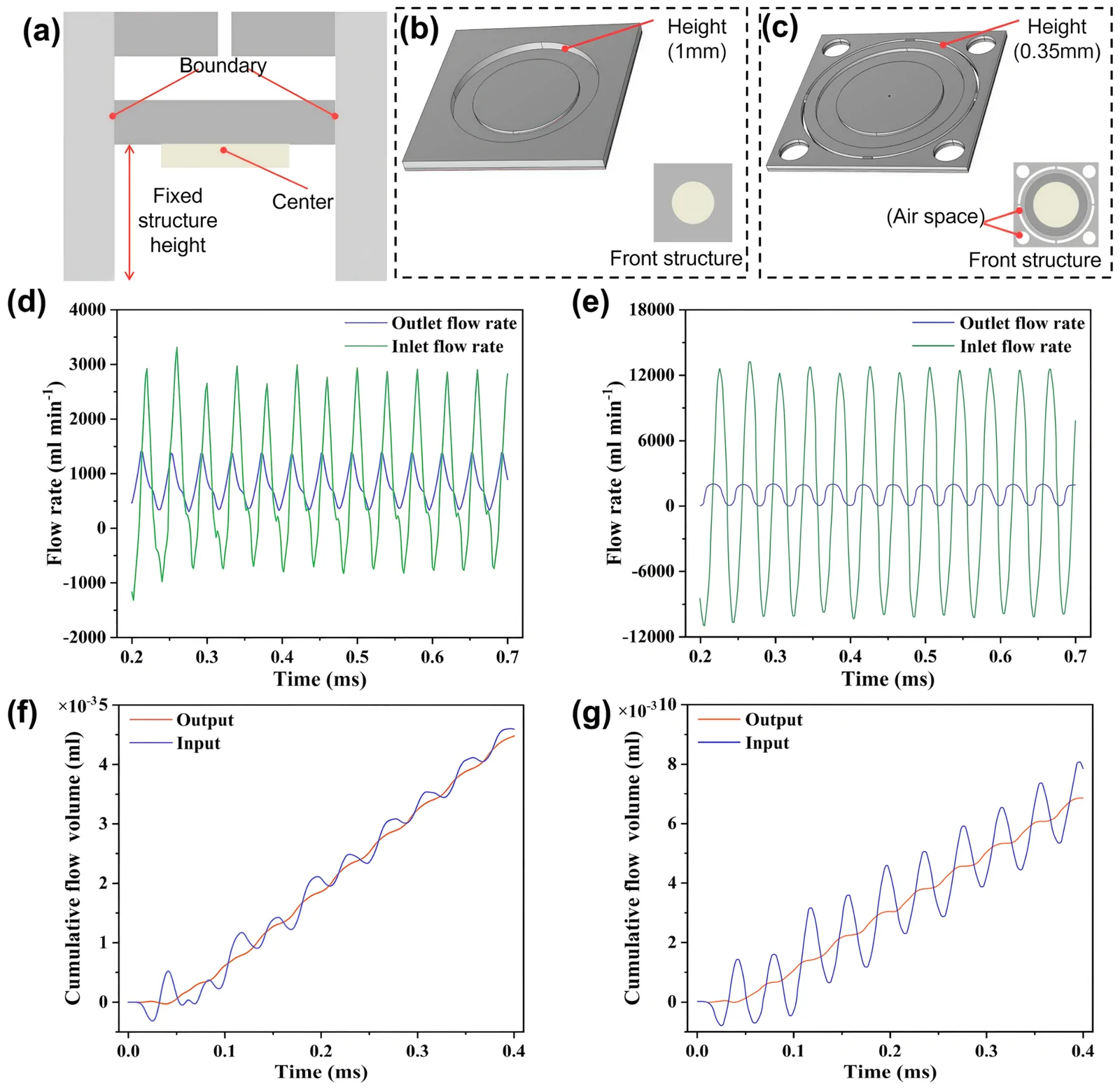
Comparison of boundary structure design and flow performance: (**a**) Two-dimensional and (**b**) three-dimensional cross-sectional sketch maps for the traditional boundary structure with additional mass blocks; (**c**) Three-dimensional sketch map for the stiffness-guided fixed boundary structure; Instantaneous flow characteristic curves for the traditional structure (**d**) and the stiffness-guided fixed boundary structure (**e**); (**f**) Cumulative flow characteristic curve of the traditional structure; (**g**) Cumulative flow characteristic curve of the stiffness-guided fixed boundary structure.

The stiffness characteristics of the boundary conditions directly affect the constraint on vibration energy, which in turn determines the rate of change in pump chamber volume and the jet output efficiency. Traditional boundary designs generally adopt a passive suppression scheme using additional mass blocks, as shown in Figure 5a,b. Although this approach can reduce the boundary vibration amplitude to some extent, it is difficult to achieve an effective balance between suppressing boundary vibration and maintaining structural compactness. Additional mass blocks significantly increase the overall mass and structural height of the device, which is inconsistent with the trend toward miniaturization and lightweight design in MEMS technology. Furthermore, the extra mechanical damping introduced by the mass blocks increases vibration energy dissipation, reducing the electromechanical energy conversion efficiency of the system.

To address the inherent limitations of the traditional boundary design, this study proposes a stiffness-guided fixed boundary structure based on active stiffness regulation, as shown in Figure 5c. This structure replaces the conventional passive suppression approach and instead constructs a lightweight, high-stiffness integrated boundary support system by collaboratively optimizing key geometric parameters, including the thickness, width, and connection configuration of the top and middle frames. This design directs vibration energy toward the effective actuation region at the vibrator center.

Quantitative comparative simulations of the output flow characteristics were conducted for the stiffness-guided fixed boundary structure and the traditional additional-mass-block structure at 35 V square-wave excitation and 26.2 kHz resonant frequency. As shown in Figure 5d,f, the average instantaneous flow rate of the traditional structure was 828.78 mL/min; however, the additional mass blocks introduced extra mechanical damping and increased the structural height, thereby limiting the miniaturization potential and energy utilization efficiency of the device.

With the stiffness-guided fixed boundary structure, boundary vibration was effectively suppressed, vibration energy was concentrated toward the center of the vibrator, the central amplitude was significantly increased, and the efficiency of pump chamber volume variation was correspondingly enhanced. The simulation results show that the average instantaneous flow rate increased to 1325 mL/min, as shown in Figure 5e,g, representing a 60% improvement over the traditional structure. Through stiffness regulation, this structure enables directional guidance of vibration energy and effectively improves the driving capability of the pump chamber without sacrificing structural compactness, providing a new theoretical foundation and engineering approach for the boundary design of high-performance miniaturized piezoelectric pumps.

### 4.3. Design of the Flow-Rectifying Channel Layer and Orthogonal Test Optimization

**Figure 6:**
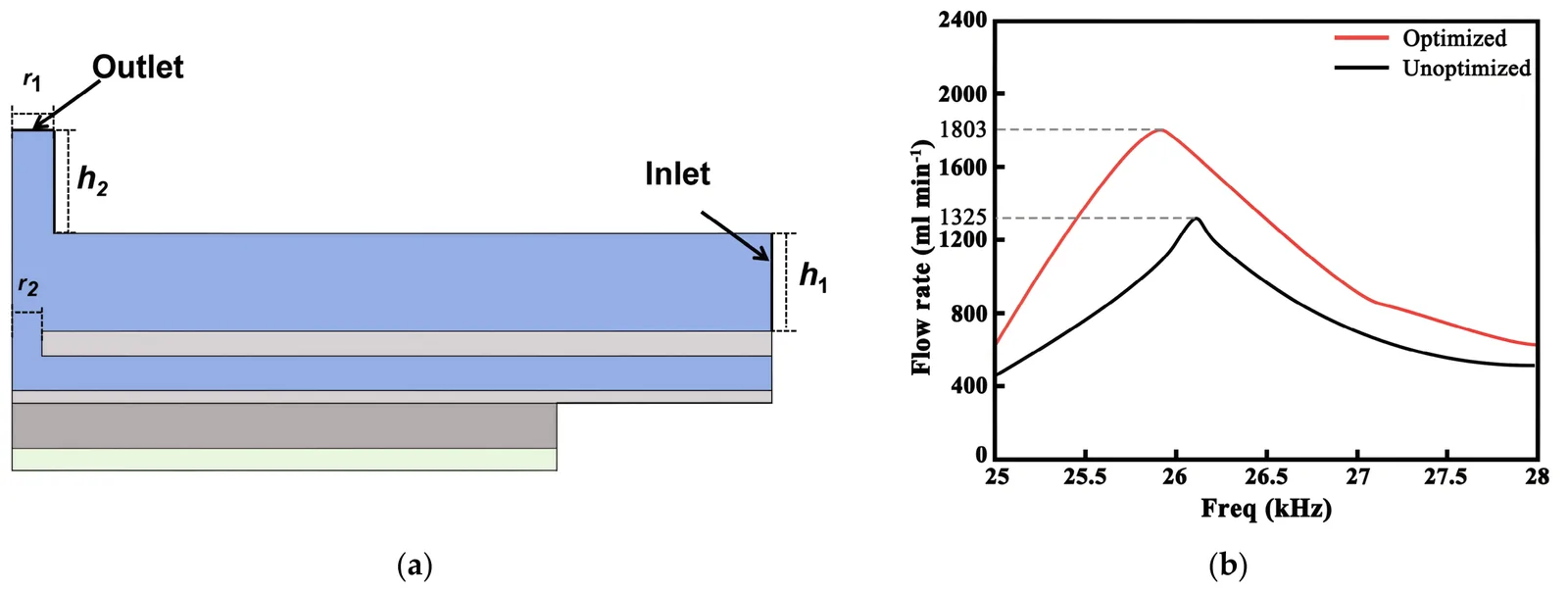
(**a**) Sketch map for the flow channel layer structure. (**b**) Comparison of output flow rate before and after optimization.

The numerical simulation results indicate that the vortex ring generated by the synthetic jet experiences viscous resistance and transverse shear from the surrounding gas during downstream propagation, resulting in a short persistence and rapid energy dissipation. To mitigate this, a dedicated flow-rectifying channel layer was introduced in the jet outlet region, as shown in Figure 6a. By rectifying and guiding the jet flow, this structure effectively suppresses the lateral shear interference of the ambient gas on the vortex ring, thereby markedly improving the structural stability and energy retention capability of the vortex ring.

For optimization of the flow channel parameters, four key geometric parameters were selected as design variables: channel outlet height (A, corresponding to h_2_), channel depth (B, corresponding to h_1_), channel radius (C, corresponding to r_1_), and jet orifice radius (D, corresponding to r_2_), as depicted in Figure 6a. To systematically assess the effect of each parameter on the output flow rate and determine the optimal parameter combination, four levels were assigned to each factor, as listed in Table 2. An L16(4^4^) standard orthogonal array was adopted for the experimental design, yielding a total of 16 test cases. Piezoelectric–fluid–structure interaction transient simulations were conducted for each parameter combination at the optimal operating frequency using COMSOL Multiphysics 6.3, and the corresponding output flow rates were recorded (Table 3).

The range analysis in Table 3 indicates that the influence of the factors follows the order: B > A > D > C. The range of channel depth B (R = 378 mL/min) is substantially greater than those of the other factors, indicating that it is the dominant parameter. In contrast, the ranges of C and D are relatively small (R = 35 and 42 mL/min, respectively), suggesting that their effects are less pronounced. The optimal level for each factor corresponds to the third level, yielding the optimal parameter combination A3B3C3D3 (A = 1.5 mm, B = 0.40 mm, C = 0.40 mm, D = 0.30 mm). This combination was not included in the original L16 orthogonal array; the closest case was No. 11 (Q = 1755 mL/min). An additional verification simulation was therefore performed, yielding a flow rate of 1803 mL/min, which was the highest among all tested cases.

As factor B increased from 0.30 mm to 0.40 mm, Q increased markedly (K_1_ → K_3_: +378 mL/min). However, when B was further increased to 0.50 mm, Q decreased to K_4_ = 1281 mL/min, indicating that the optimal channel depth is approximately 0.40 mm. Factor A exhibited optimal performance at 1.5 mm; a further increase to 1.8 mm resulted in reduced performance. Figure 6b compares the output flow rates before and after optimization. With the optimal parameter combination, the output flow rate increased from 1325 mL/min to 1803 mL/min, corresponding to an increase of 36.1%, as shown in Table 4.

## 5. Device Preparation and Experimental Performance Testing

### 5.1. Device Manufacturing and Test System Construction

**Figure 7:**
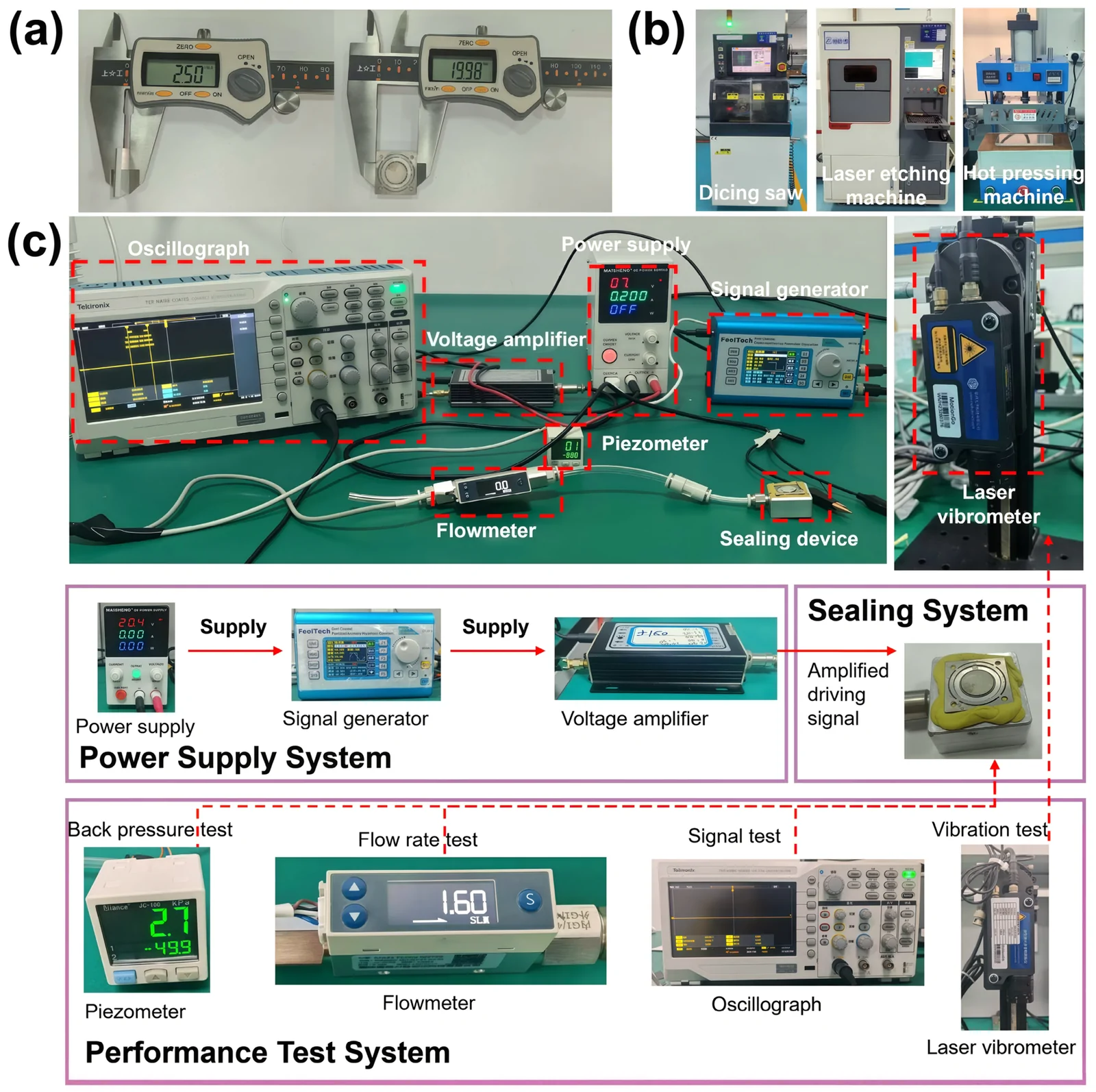

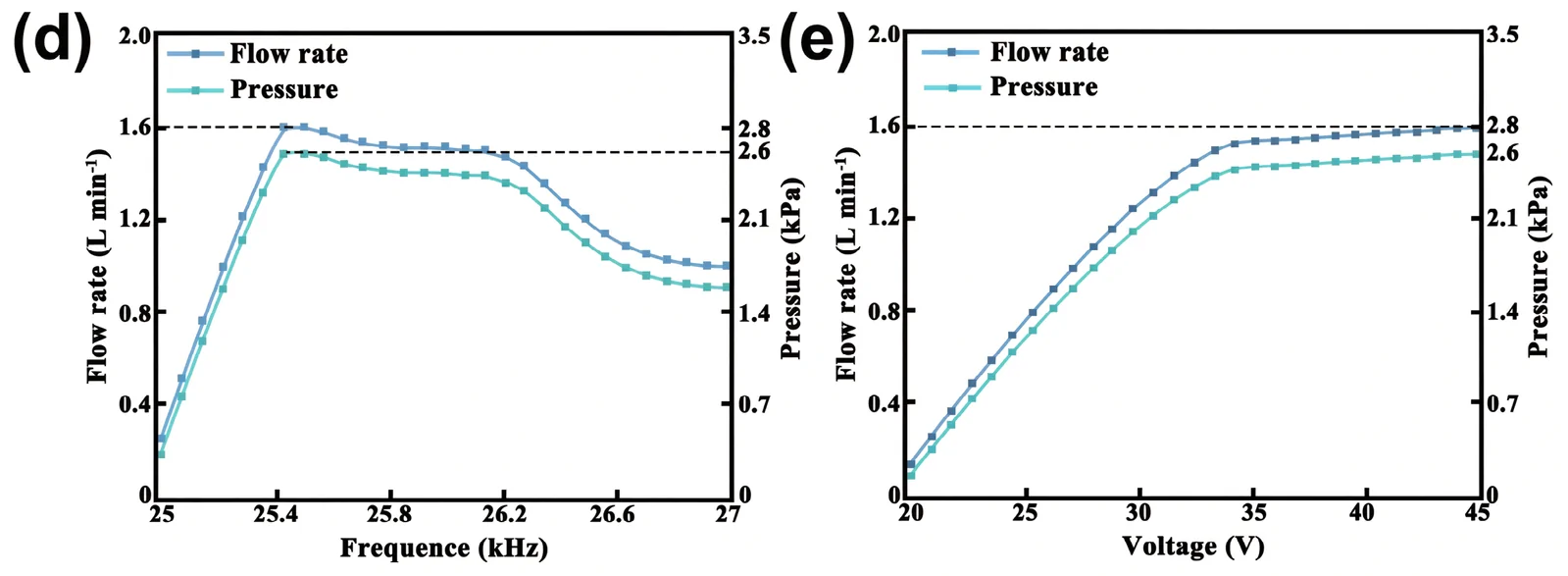
Piezoelectric pump prototype and performance testing: (**a**) Piezoelectric pump prototype; (**b**) Equipment used for piezoelectric pump fabrication; (**c**) Piezoelectric pump testing system; (**d**) Frequency-flow rate and frequency-back pressure characteristic curves; (**e**) Voltage-flow rate and voltage-back pressure characteristic curves.

To comprehensively verify the effectiveness of the proposed ultrasonic synthetic jet piezoelectric pump and its optimization strategy, device fabrication and system performance testing were carried out. Based on the optimized simulation results, the piezoelectric pump was fabricated using a metal-based non-silicon MEMS process. The key components, including the top frame, piezoelectric vibrator, middle-frame resonant layer, and flow channel layer, were assembled through precise alignment and bonding processes. The overall dimensions of the device were 20 mm × 20 mm × 2.5 mm, demonstrating a high degree of integration and miniaturization, as shown in Figure 7a. The core fabrication equipment is shown in Figure 7b, including a laser etching machine, a hot-press machine, and a dicing saw, which were used for precision machining of the key structural layers, hot-press bonding of the multilayer materials, and separation of the final device units, respectively.

The experimental test system is shown in Figure 7c. It consisted of a high-frequency signal generator, a voltage amplifier, a precision gas flowmeter, a micro-differential pressure sensor, and a laser vibrometer. The system enabled accurate measurement of the output flow rate, back pressure, and vibration characteristics of the piezoelectric pump at room temperature. The specifications of the measurement instruments are summarized in Table 5.

### 5.2. Frequency and Voltage Response Characteristics

Under 35 V square-wave excitation, the piezoelectric pump achieved optimal output performance at the 25.5 kHz resonant frequency, delivering an outlet flow rate of 1.6 L/min and a back pressure of 2.7 kPa. The experimental values were slightly lower than the simulated predictions, which can be attributed to the following factors: (1) unavoidable dimensional tolerances during MEMS fabrication (on the order of ±5 μm), resulting in deviations in the actual flow channel geometry from the design values; (2) alignment errors and additional contact damping introduced during the bonding and assembly of the multilayer structure; and (3) incomplete consideration of the nonlinear losses of the piezoelectric materials and the compressibility effects of air in the simulation model. Figure 7d presents the frequency–flow rate and frequency–back pressure characteristics of the device. The pump exhibited stable output performance in the vicinity of the resonant frequency.

Quantitatively, the experimental flow rate (1.6 L/min) is approximately 11.3% lower than the numerically predicted optimal value (1803 mL/min). This discrepancy can be primarily attributed to the three factors discussed above. Each data point reported in this study represents the average of four independent measurements, with a standard deviation of less than 3%. No observable gas leakage was detected during the experiments. All instruments were calibrated prior to measurement in accordance with standard procedures.

The influence of driving voltage on output performance was further analyzed. Figure 7e presents the voltage–flow rate and voltage–back pressure characteristics. Both output flow rate and back pressure increased monotonically with driving voltage. However, when the driving voltage exceeded 35 V, significant heating of the device was observed. Prolonged operation under such conditions increases the risk of depolarization of the piezoelectric ceramics and may result in performance degradation. Therefore, 35 V (70 Vpp) was selected as the optimal operating voltage in the present work to balance output performance and long-term device reliability.

### 5.3. Comprehensive Performance Comparison with Related Studies

To objectively evaluate the contribution of this work, a comprehensive comparison was conducted between the proposed piezoelectric pump and previously reported devices, as summarized in Table 6. At a driving voltage of 70 Vpp, an operating frequency of 25.5 kHz, and an ultrathin structural height of 2.5 mm, the proposed device achieved a flow output of 1.6 L/min. Its overall performance surpasses that of most comparable micropumps reported in the literature. The primary advantage of this work lies in achieving high output performance per unit volume under a low driving voltage and within a highly compact structure, making the device particularly suitable for low-power, portable applications with limited installation space.

## 6. Conclusions

This study systematically investigated the vibration performance of the piezoelectric vibrator through numerical simulation and analyzed the evolution mechanism of the synthetic jet flow field. A high-performance ultrasonic synthetic jet piezoelectric pump was designed and fabricated, showing potential for heat dissipation applications in high-power-density microelectronics. Through multi-level structural innovation and multiphysics collaborative optimization, this study addressed the technical bottlenecks of traditional synthetic jet piezoelectric pumps, including limited flow output under low-voltage driving, narrow operating bandwidth, and low energy conversion efficiency. Under 35 V square-wave excitation, the device achieved a maximum output flow rate of 1.6 L/min and a maximum back pressure of 2.7 kPa within an ultra-compact package size of 20 mm × 20 mm × 2.5 mm. The comprehensive performance indicators of the proposed device are competitive with the state of the art. Future studies will focus on comprehensive efficiency characterization, full pressure–flow curve measurement, and multi-objective optimization of the pump.

The core findings of this study are as follows:

1. Systematic clarification of vibration and flow mechanisms: Through finite-element modal simulation and transient fluid–structure interaction analysis, the vibration characteristics and energy distribution patterns of each mode of the piezoelectric vibrator were clarified, and the sixth-order axisymmetric resonant mode (26.2 kHz) was selected as the operating mode. The formation, development, and transport mechanisms of the synthetic jet vortex ring over a complete operating cycle were fully elucidated, verifying the feasibility of stable synthetic jet formation with the proposed structure.
2. Dual-resonant-layer structure design: A resonant lower-frame structure was introduced, and a coupled pump chamber system featuring coordinated vibration between the piezoelectric vibrator and the resonant layer was developed. Theoretical analysis and simulation verification demonstrated that the out-of-phase vibration mode increased the maximum chamber volume change rate by 21% and the output flow rate by 36.4%.
3. Innovation of the stiffness-guided fixed boundary structure: A stiffness-guided fixed boundary structure based on active stiffness regulation was proposed. By synergistically optimizing the geometric parameters of the upper and middle frames, a lightweight, high-stiffness integrated support system was constructed, which directed vibration energy toward the effective actuation region at the vibrator center and significantly increased the central amplitude. Simulation results demonstrated that this structure further increased the output flow rate by 60%, reaching 1325 mL/min.
4. Flow-rectifying channel layer design and orthogonal test optimization: A dedicated flow-rectifying channel layer was added to the jet outlet region, effectively suppressing the lateral shear interference of the ambient gas on the vortex ring. Four key channel parameters were optimized using an L16(4^4^) orthogonal test design, and the optimal parameter combination (A = 1.5 mm, B = 0.40 mm, C = 0.40 mm, D = 0.30 mm) was determined through range analysis. After optimization, the output flow rate increased from 1325 mL/min to 1803 mL/min, representing a 36.1% improvement, which significantly enhanced the directional transport capability of the synthetic jet.

It should be noted, however, that the thermal management performance of the proposed pump has not yet been experimentally validated through dedicated cooling tests. No heater temperature, thermal resistance, heat-transfer coefficient, or removable heat load data have been reported in this study. The current discussion regarding thermal management applications is based solely on the pump’s airflow delivery capability. Further cooling experiments with representative heat sources are required to quantitatively evaluate the actual heat dissipation performance and validate the thermal-management potential of this device.

## Figures and Tables

**Table 1:** Material properties of the piezoelectric vibrator components.

Material	Density (kg/m^3^)	Young’s Modulus (GPa)	Poisson’s Ratio	d_33_ (pC/N)
PZT	7500	81.3	0.33	289
SS304	7936	191.5	0.29	—
SS430	7866	220	0.29	—

**Table 2:** Level values of flow channel parameters.

Parameter Symbol	Parameter Name	Level 1	Level 2	Level 3
A	Channel height (mm)	1.0	1.3	1.5
B	Channel depth (mm)	0.30	0.35	0.40
C	Channel radius (mm)	0.30	0.35	0.40
D	Jet orifice radius (mm)	0.20	0.25	0.30

**Table 3:** L16(4^4^) orthogonal test scheme, results, and range analysis.

No.	A (mm)	B (mm)	C (mm)	D (mm)	Q (mL/min)
1	1.0	0.30	0.30	0.20	1152
2	1.0	0.35	0.35	0.25	1376
3	1.0	0.40	0.40	0.30	1602
4	1.0	0.50	0.50	0.40	1160
5	1.3	0.30	0.35	0.30	1327
6	1.3	0.35	0.30	0.40	1448
7	1.3	0.40	0.50	0.20	1656
8	1.3	0.50	0.40	0.25	1320
9	1.5	0.30	0.40	0.40	1381
10	1.5	0.35	0.50	0.30	1584
11	1.5	0.40	0.30	0.25	1755
12	1.5	0.50	0.35	0.20	1380
13	1.8	0.30	0.50	0.25	1260
14	1.8	0.35	0.40	0.20	1454
15	1.8	0.40	0.35	0.40	1621
16	1.8	0.50	0.30	0.30	1263
K_1_	1322	1280	1404	1410	
K_2_	1438	1466	1426	1428	
K_3_	1525	1658	1439	1444	
K_4_	1400	1281	1415	1402	
R	202	378	35	42	
Optimal level	A_3_ (1.5)	B_3_ (0.40)	C_3_ (0.40)	D_3_ (0.30)	

Note: K_i_ represents the mean Q value (mL/min) corresponding to the i-th level of each factor, and R denotes the range.

**Table 4:** Parameters and performance before and after optimization.

Scheme	A (mm)	B (mm)	C (mm)	D (mm)	Q (mL/min)
Initial scheme	1.7	0.3	0.4	0.3	1325
Optimal scheme	1.5	0.4	0.4	0.3	1803

**Table 5:** Specifications of the measurement instruments.

Instrument	Model	Range	Accuracy	Manufacturer
Signal Generator	FeelTech FY6900	0.1 μHz–30 MHz 1 mVpp–24 Vpp	±20 ppm (freq.) ±1% F.S. (ampl.)	FeelElec, Zhengzhou, China
Laser Vibrometer	Polytec CLV-2534	0.5 Hz–3.2 MHz ± 10 m/s	<0.1 nm (displ.) 0.05 μm/s (vel.)	Polytec GmbH, Waldbronn, Germany
Gas Flowmeter	SMC PF2M710	0.1–10 L/min (air)	±3% F.S. ± 1 digit	SMC Corporation, Tokyo, Japan
Pressure Sensor	SMC ZSE40A	−100 to +100 kPa	±0.2% F.S. ± 1 digit 0.1 kPa (resol.)	SMC Corporation, Tokyo, Japan

**Table 6:** Comparison of piezoelectric pump performance with those reported in previous studies.

Report	Vpp (V)	Frequency (kHz)	Size	Height (mm)	Flow (L/min)
Wang	80	20.4	30 mm × 30 mm	5	0.39
Liu	150	2.38	Φ55 mm/Φ11.5 mm	5/11.5	2.1/2.43
Li	100	3.7	Φ28 mm	10	3
Wang	60	20.65	Φ30 mm	5.5/6.5	1.34/1.91
This article	70	25.5	20 mm × 20 mm	2.5	1.6

## Data Availability

All key performance data validating the conclusions are provided in the article. The original contributions presented in the study are included in the article. Further inquiries can be directed to the corresponding author.

## Abbreviation

MEMS	Micro-electro-mechanical system
